# Dynamic modelling of cold-hardiness in tea buds by imitating past temperature memory

**DOI:** 10.1093/aob/mcaa197

**Published:** 2020-11-28

**Authors:** Kensuke Kimura, Daisuke Yasutake, Takahiro Oki, Koichiro Yoshida, Masaharu Kitano

**Affiliations:** 1 National Agriculture and Food Research Organization (NARO), Institute of Agro-Environmental Sciences, Kannondai, Tsukuba, Ibaraki, Japan; 2 Kyushu University, Faculty of Agriculture, Fukuoka, Japan; 3 Kyushu University, Graduate School of Bioresource and Bioenvironmental Sciences, Fukuoka, Japan; 4 Kochi University, Faculty of Agriculture and Marine Science, Kochi, Japan

**Keywords:** Cold-hardiness, plant memory, acclimation, abiotic stress, cold stress, mathematical model, seasonal variation, *Camellia sinensis*

## Abstract

**Background and Aims:**

Most perennial plants memorize cold stress for a certain period and retrieve the memories for cold acclimation and deacclimation, which leads to seasonal changes in cold-hardiness. Therefore, a model for evaluating cold stress memories is required for predicting cold-hardiness and for future frost risk assessments under warming climates. In this study we develop a new dynamic model of cold-hardiness by introducing a function imitating past temperature memory in the processes of cold acclimation and deacclimation.

**Methods:**

We formulated the past temperature memory for plants using thermal time weighted by a forgetting function, and thereby proposed a dynamic model of cold-hardiness. We used the buds of tea plants (*Camellia sinensis*) from two cultivars, ‘Yabukita’ and ‘Yutakamidori’, to calibrate and validate this model based on 10 years of observed cold-hardiness data.

**Key Results:**

The model captured more than 90 % of the observed variation in cold-hardiness and predicted accurate values for both cultivars, with root mean square errors of ~1.0 °C. The optimized forgetting function indicated that the tea buds memorized both short-term (recent days) and long-term (previous months) temperatures. The memories can drive short-term processes such as increasing/decreasing the content of carbohydrates, proteins and antioxidants in the buds, as well as long-term processes such as determining the bud phenological stage, both of which vary with cold-hardiness.

**Conclusions:**

The use of a forgetting function is an effective means of understanding temperature memories in plants and will aid in developing reliable predictions of cold-hardiness for various plant species under global climate warming.

## INTRODUCTION

Seasonal changes in cold-hardiness due to cold acclimation and deacclimation processes are important mechanisms that enable plants to prevent new and vulnerable tissues from being damaged by frost, which is common in agriculture and economically detrimental (Snyder *et al.*, 2005). Furthermore, as cold acclimation and deacclimation are mainly driven by temperature, these processes are strongly affected by global climate change (Pagter and Arora, 2013). According to the IPCC (2014), the mean surface air temperature will increase in the future, and frequent extreme temperature events will lead to more frequent acclimation and deacclimation cycles (Vitasse *et al.*, 2014). In particular, shorter and milder winters and an increase in the number of warm spells in the spring will result in insufficient cold acclimation and an earlier start to the deacclimation process in the growing season, increasing the risk of frost damage (Augspurger, 2013; Richardson *et al.*, 2018). There have been many previous investigations into the risks of frost damage in past and future climates, using a variety of approaches. Robeson (2002) investigated trends in spring and autumn (fall) freezing events in 1906–97 using observed daily minimum air-temperature data, while Marino *et al.* (2011) estimated the risk of frost damage in 1901–2007 by comparing phenology (the timing of the start of the growing season) data and minimum temperature (the timing of a potentially damaging freeze) data. However, the risk of frost damage in past and future climates should be evaluated when considering the seasonal changes in cold-hardiness via the processes of cold acclimation and deacclimation, because frost damage develops when the temperature of plant tissues drops below their cold-hardiness limitations. The use of cold-hardiness to estimate frost damage has not previously been widely researched, owing to the difficulties in obtaining frequent and long-term measurements.

Model simulations remain the sole approach for long-term analysis of cold-hardiness, with some having already been developed for Scots pine (Leinonen, 1996), cereals (Fowler *et al*., 1996), winter wheat (Bergjord *et al.*, 2008) and grapevine (Ferguson *et al.*, 2011). Conventional models often use daily temperatures that are below threshold temperatures (thermal time) to estimate daily changes in cold-hardiness, and this indicates that plants increase cold-hardiness from the previous day to the current day by referring only to the previous day’s temperature. However, this may not reflect the recent finding of ‘plant memory’, whereby plants respond as if they learn, store and recall environmental stimuli over certain periods. This involves a complex molecular network involving calcium waves, epigenetic modifications of DNA and histones, and regulation of timing via a biological clock (Thellier and Lüttge, 2013; Demongeot *et al.*, 2019), although plants do not have neurons, which are responsible for memory functions in animals (for reviews on plant memory, please see Trewavas, 2003, Thellier and Lüttge, 2013; Crisp *et al*., 2016). In the flowering phenology of the perennial *Arabidopsis halleri*, as much as 83 % of seasonal expression variation of the temperature-dependent flowering-time gene is explained solely by accumulated temperatures over the previous 6 weeks (Aikawa *et al.*, 2010). During cold acclimation, plants are primed with a first stress experience of cold to improve their responses to subsequent stresses, and they memorize repeated stresses to increase their cold-hardiness (Baier *et al.*, 2019). In deacclimation processes, plants forget the cold stress memory and thus lose their cold-hardiness upon exposure to warmer temperatures in the spring, to resume growth and development (Crisp *et al.*, 2016; Vyse *et al.*, 2019). However, how long and how much past temperatures affect cold acclimation and deacclimation remain unclear, as massive reorganizations in gene expression and metabolism are related to cold acclimation and deacclimation (Thomashow, 1999; Wanner and Junttila, 1999; Baier *et al.*, 2019). Currently, excluding a model using temperatures averaged over the last several days (Charrier *et al.*, 2018*b*), there is no cold-hardiness model that considers the temperature memories of plants when determining cold acclimation and deacclimation processes.

The tea plant (*Camellia sinensis*) is one of the most important economic crops in many parts of the world (Wang *et al.*, 2013), but it has been severely damaged by frost in recent years. Consequently, economic losses in tea production due to frost damage reached 268 million USD in 2010 in China (China Tea Marketing Association, 2010), and 45 million USD in 2010 in Japan (Matsuo *et al.*, 2010). It is thus imperative that we develop accurate prediction methods for cold-hardiness, to efficiently operate frost protection systems and prevent frost damage in tea plants. Although tea plants enhance their cold-hardiness with decreased temperatures and conversely reduce it with increased temperatures, similar to other perennial woody crops, there is no predictive model currently available for determining the cold-hardiness of tea plants.

We developed a new dynamic model of cold-hardiness by introducing a function imitating past temperature memory in the processes of cold acclimation and deacclimation. The developed model has been parameterized for two tea plant cultivars and validated using 10 years of cold-hardiness data measured in field conditions, using 2-fold cross-validation to increase model reliability.

## MATERIALS AND METHODS

### Measuring cold-hardiness

Tea bud samples (*n* = 15) were collected from tea fields located in Kagoshima, Japan (Kagoshima Prefectural Institute for Agricultural Development; 31°22′02″ N, 130°26′57″ E, 175 m a.m.s.l.). The following two cultivars were sampled from autumn (November) to spring (April) for 10 years, at intervals of ~1 week: ‘Yabukita’ (2008–18), which is widely cultivated in Japan; and ‘Yutakamidori’, which sprouts earlier than ‘Yabukita’ (2008–19, except 2009–10 and 2017–18).

Controlled freezing tests were conducted to quantify the cold-hardiness of the tea buds. The samples were each equally separated into three subsamples, and each subsample was placed in a freezer (EC-15MTP, Hitachi, Tokyo, Japan), which was programmed to progressively drop its temperature. The subsamples were first held at 0 °C in the freezer, and then frozen at a rate of 1 °C h^−1^ until specified target temperatures were reached, which were differently assigned to each subsample, at intervals of 1 °C. The target temperatures were determined according to their sampling dates; for example, the temperatures were −1, −2 and −3 °C in early autumn and −14, −15 and −16 °C in midwinter. After reaching the target temperature, each subsample was stored for 3 h at the temperature, and was then thawed at a rate of 3 °C h^−1^ until 5 °C. After thawing, all subsamples were stored at 5 °C for 2 d. Subsequently, the samples were removed from the freezer and dissected to determine tissue damage using a visual scoring method (Lindgren and Hallgren, 1993). With this method, the tissue damage of each sample was assessed for discoloration of the dissected sample, and the discoloration was scored in four classes based on the percentage of the whole buds that were occupied by discoloured tissue: 0, 0 % discoloration; 0.25, 1–33 % discoloration; 0.5, 34–66 % discoloration; and 1, 67–100 % discoloration. Using this scoring system, the percentage of dead samples in each subsample (*P*) was assessed as follows:

$$\begin{array}{l} P\;=\;\frac{n_{0}\times 0+n_{0.25}\times 0.25+n_{0.5}\times 0.5+n_{1}\times 1\;}{n}\times 100 \end{array}$$ (1)

where *n* is the number of samples and the subscripts represent the discoloration scores. In this study lethal temperatures caused damage to 10 % of the samples (LT_10_). The temperature at which *P* was not >10 % was used as a quantitative indicator of cold-hardiness. Other modelling studies in tree cold-hardiness have used LT_50_, at which 50 % of buds are killed, but 50 % damage would be fatal for agricultural practices. Thus, we used LT_10_ to predict the minimum frost damage in actual crop production.

### Model description

Cold-hardiness, expressed as LT_10_ for the tea buds, at the current time *t* (LT_10,*t*_) was estimated from the previous day’s value (LT_10,*t*−1_) and the change in hardiness (*∆*LT_10_).

$$\begin{array}{l} LT_{10,t}\;=\;LT_{10,t-1}\;+\;\Delta LT_{10} \end{array}$$ (2)

We calculated *∆*LT_10_ from the mean daily air temperature in each process of cold acclimation and deacclimation.

**Fig. 1.**
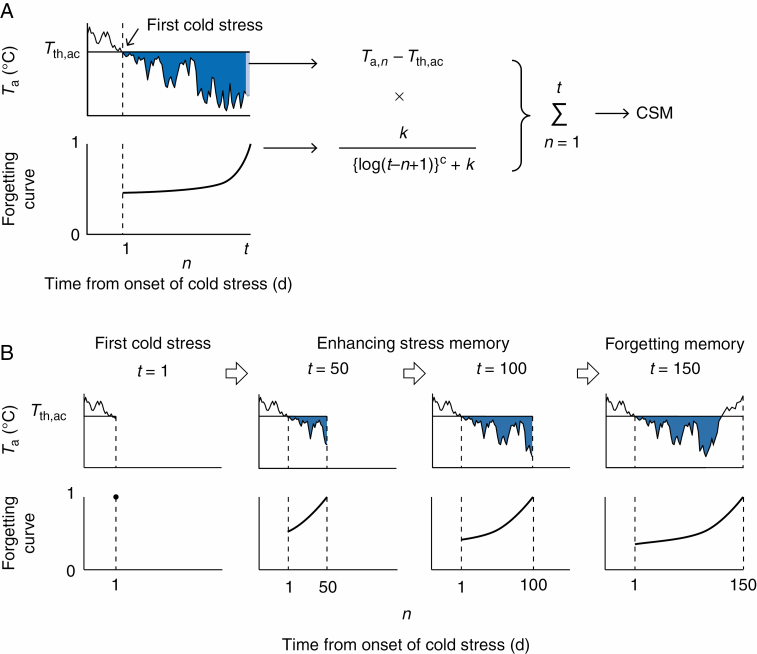
Concept of (A) the function of cold stress memory (CSM) that is expressed by thermal time below the threshold temperature (*T*_th,ac_) and the forgetting curve from the first cold stress, and (B) time courses of CSM updated on a daily basis.

During cold acclimation, *∆*LT_10_ was expressed as the difference in effective accumulated temperature for cold acclimation (Σ*T*_ac_) between the current and previous days.

$$\begin{array}{l} \Delta LT_{10}\;=\;\left(\sigma T_{ac,t}-\sigma T_{ac,t-1}\right)\;\times \;H_{ac}\;\times \;S \end{array}$$ (3)

where *H*_ac_ is the acclimation rate constant and *S* is the function representing the slowdown of the acclimation rate. The value of σ*T*_ac_ is given by the function representing cold stress memory (CSM), which is expressed as the accumulation of thermal time, weighted by the forgetting curve, which was originally developed by Ebbinghaus (1885/2013) for research into human memory:

$$\begin{array}{l} \sigma T_{ac,t}\;=\;CSM_{t}\; \end{array}$$ (4)

$$\begin{array}{l} CSM_{t}\;=\sum_{n\;=1}^{t}\left\{\left(T_{a,n}-T_{th,ac}\right)\;\times \;\frac{k}{{\left\{log\left(t-n\;+\;1\right)\right\}}^{c}\;+\;k}\right\} \end{array}$$ (5)

where *T*_a_ is the daily mean air temperature, *T*_th,ac_ is the threshold temperature effective in cold acclimation, and *k* and *c* are the parameters of the forgetting curve. The forgetting curve shows values from 0 to 1, and the values are highest on the latest day and decrease gradually as the number of days decreases (Fig. 1A). Thus, eqn (5) shows that the thermal time in the later days (*n* close to *t*) strongly contributes to cold acclimation and that in the early days (*n* close to 1) it does not (Fig. 1A), and the forgetting function was applied similarly to determine the accumulation period of the thermal time. The calculation of eqn (5) began when *T*_a_ was first below *T*_th,ac_ in the autumn (Fig. 1B), and this represents the process of cold priming, when plants prepare to improve their cold-hardiness as they experience their first cold stress (Baier *et al.*, 2019). Additionally, eqn (5) shows that plants enhance their cold memory by experiencing repeated cold stresses (Crisp *et al.*, 2016) (Fig. 1B). In summary, eqn (5) imitates the current plant memory for cold acclimation, and this memory is updated daily. The slowdown of cold acclimation in midwinter was commonly observed in many species (Brule-Babel and Fowler, 1989; Fowler *et al.*, 1996; Ferguson *et al.*, 2011), and the model assumed that the cold acclimation rate gradually slowed as CSM approached its critical level (CSM_ac_), and this was expressed by the function *S*.

$$\begin{array}{l} \;S\;=\;1-\frac{CSM_{t-1}}{CSM_{ac}}\;\left(CSM_{t-1}\;>\;CSM_{ac}\right) \end{array}$$ (6)

$$\begin{array}{l} \;S\;=\;0\;\left(CSM_{t-1}\;\leq \;CSM_{ac}\right) \end{array}$$

The transition from cold acclimation to deacclimation is related to the phenological stage of the bud, and this depends on the transition from an endodormant to an ecodormant state (Vyse *et al.*, 2019). Many perennial species require a period of exposure to cold temperatures (chilling requirements) to transition from endodormancy to ecodormancy (Charrier *et al.*, 2011). Additionally, some temperate and subtropical species become sensitive to photoperiods when chilling requirements are not met (Laube *et al.*, 2014; Du *et al.*, 2019), i.e. insufficient chilling can be offset by a longer photoperiod (Du *et al.*, 2019). With consideration of these hypotheses, transitions from cold acclimation to deacclimation, in this model, were assumed to occur when CSM reached its critical level (CSM_de_) or the photoperiod reached its critical length (*P*_de_).

During deacclimation, *∆*LT_10_ is expressed as the difference in the effective accumulated temperatures to deacclimation (σ*T*_de_) between the current and previous day.

$$\begin{array}{l} \Delta LT_{10}\;=\;\left(\sigma T_{de,t}-\sigma T_{de,t-1}\right)\;\times \;H_{de} \end{array}$$ (7)

where *H*_de_ is the deacclimation rate constant. σ*T*_de_ is expressed as competition between CSM, which represents forgetting the cold stress memory in warm temperatures or reacquiring the memory in response to sudden cold temperatures, and the function related to acceleration of deacclimation in response to warm temperatures (GDD), which is expressed as the accumulation of thermal time above the threshold temperature (*T*_th,de_).

$$\begin{array}{l} \sigma T_{de,t}\;=\;CSM_{t}\;+\;GDD_{t} \end{array}$$ (8)

$$\begin{array}{l} GDD_{t}\;=\sum_{n\;=\;de}^{t}\left(T_{a,n}-T_{th,de}\right) \end{array}$$ (9)

where de represents the start of deacclimation. In warm temperatures both CSM and GDD increased, and deacclimation was markedly accelerated. However, when the temperatures suddenly dropped again during deacclimation, CSM decreased beyond the increase in GDD and the lost cold-hardiness was regained. This process is called reacclimation (Kovi *et al.*, 2016).

### Input meteorological data

The daily mean air temperature *T*_a,*t*_ was obtained from the Agro-Meteorological Grid Square Data, which provides 1-km^2^-gridded data on meteorological variables using spatial interpolation among the observed values at meteorological stations in Japan, and corrects for the differences in temperature due to elevation (Ohno *et al.*, 2016). The values for the squares, including those for the experimental tea field, were used in this investigation. Photoperiods were calculated using the day of year, sun position and latitude of the tea field.

### Model parameterization and validation

The initial value of LT_10_ (LT_10,ini_) was set at −2 °C, which is the maximum measured value of LT_10_ without cold acclimation and deacclimation. Other model parameters (*H*_ac_, *T*_th,ac_, *k*, *c*, CSM_ac_, CSM_de_, *P*_de_, *H*_de_, *T*_th,de_) were assessed by minimization of the root mean square error (RMSE) between the estimated values and the observed data on LT_10_. For parameterization, the differential evolution algorithm was applied with the DEoptim R package (version 2.2–5), as proposed by Mullen *et al.* (2011). For model validation, 2-fold cross-validation was used. The dataset was randomly partitioned into two sets (*d*_1_ and *d*_2_), and then the model was calibrated on *d*_1_ and validated on *d*_2_, followed by calibrating on *d*_2_ and validating on *d*_1_. The optimized parameters using the complete data set (10 years of data) are shown in Table 1.

**Table 1.** Model parameters used to simulate cold-hardiness in the tea cultivars ‘Yabukita’ and ‘Yutakamidori’

Parameter	Description	Value		Unit	Fixed or fitting
		‘Yabukita’	‘Yutakamidori’		
LT_10,ini_	Initial cold-hardiness	−2	−2	°C	Fixed
*T*_th,ac_	Threshold temperature effective for cold acclimation	17.4	16.1	°C	Fitting
*H*_ac_	Acclimation rate constant	0.051	0.038	°C °C^−1^	Fitting
CSM_ac_	Critical cold-stress memory affecting the slowdown of the cold acclimation rate	−489.8	−594.3	°C	Fitting
*k*	Parameter of the forgetting curve	18.7	10.6	Dimensionless	Fitting
*c*	Parameter of the forgetting curve	3.4	2.5	Dimensionless	Fitting
*T*_th,de_	Threshold temperature related to acceleration of deacclimation	−4.0	−17.1	°C	Fitting
*H*_de_	Deacclimation rate constant	0.032	0.015	°C °C^−1^	Fitting
CSM_de_	Critical cold-stress memory indicating that tea buds are ready for deacclimation	−605	−491	°C	Fitting
*P*_de_	Critical day length for the start of deacclimation	661	654	min	Fitting

### Sensitivity analysis

To evaluate the sensitivity of the fitted parameters for each cultivar, a sensitivity analysis was completed for all fitted parameters within the developed model. The optimized value of a parameter, as shown in Table 1, was changed at 1 % intervals from –5 to + 5 %, whereas all other parameters were maintained at their optimized values. The RMSE between the simulated and observed values of LT_10_ over 10 years was used as the output variable.

The normalized sensitivity coefficient (NSC) was used to quantify the sensitivity of the model to each parameter and was defined as the ratio between the percentage changes in RMSE (*∆*RMSE/RMSE_opt_) and the percentage changes in parameter values (*∆P*/*P*_opt_):

$$\begin{array}{l} \;NSC\;=\;\frac{\Delta RMSE\;/\;RMSE_{opt}}{\Delta P\;/\;P_{opt}} \end{array}$$ (10)

where RMSE_opt_ is the RMSE under optimized parameter values and *∆*RMSE is the change in RMSE under the changed parameter values in comparison with RMSE_opt_. The coefficients over the entire range of the percentage changes for each parameter were averaged to evaluate mean normalized sensitivity coefficients (NSC_mean_).

## RESULTS

### Parameter values

**Fig. 2.**
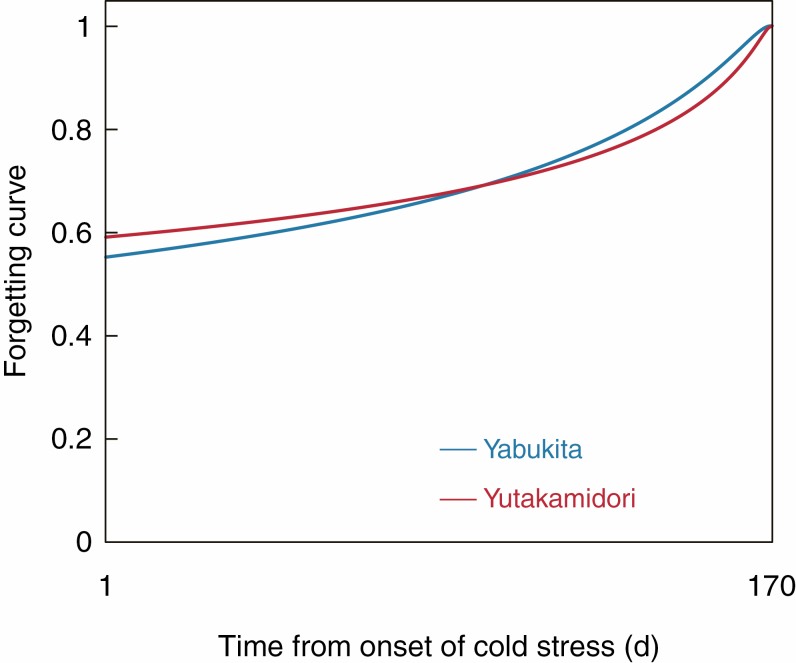
Forgetting curves optimized for ‘Yabukita’ and ‘Yutakamidori’ to simulate cold-hardiness. Curves from 1 to 170 d after the onset of cold stress are shown as an example. The optimized parameters in the curve are described in Table 1.

The optimized, cultivar-specific parameters of the model are listed in Table 1. ‘Yabukita’ starts its cold acclimation earlier, and it is more rapid from autumn to winter than that of ‘Yutakamidori’. These characteristics were reflected by higher *T*_th,ac_ and *H*_ac_ values in ‘Yabukita’. Optimized forgetting curves, including the parameters *k* and *c*, are shown in Fig. 2. The curves for both ‘Yabukita’ and ‘Yutakamidori’ showed high values (near 1) in recent days and approximately half values (near 0.6) at the start of cold acclimation. The forgetting curve for ‘Yabukita’ has a larger inclination than that of ‘Yutakamidori’, which indicates that ‘Yabukita’ is relatively more sensitive to recent cold stresses than ‘Yutakamidori’. In the model developed here, the forgetting curve and the threshold temperatures *T*_th,ac_ determined CSM in the tea buds that regulated cold acclimation rates, and CSM was further used as the index to decelerate the acclimation rate in midwinter (CSM_ac_), and the cue to start deacclimation (CSM_de_). With higher CSM_ac_, ‘Yabukita’ showed an earlier slowdown of the acclimation rate and developed maximum cold-hardiness earlier than ‘Yutakamidori’ in midwinter. With lower CSM_de_ and longer *P*_de_, ‘Yabukita’ required stronger cold stress and longer day lengths to start the deacclimation than ‘Yutakamidori’. Despite the higher *H*_de_, ‘Yabukita’ showed slower rates of deacclimation than ‘Yutakamidori’, owing to the markedly higher *T*_th,de_.

### Model validation

**Fig. 3.**
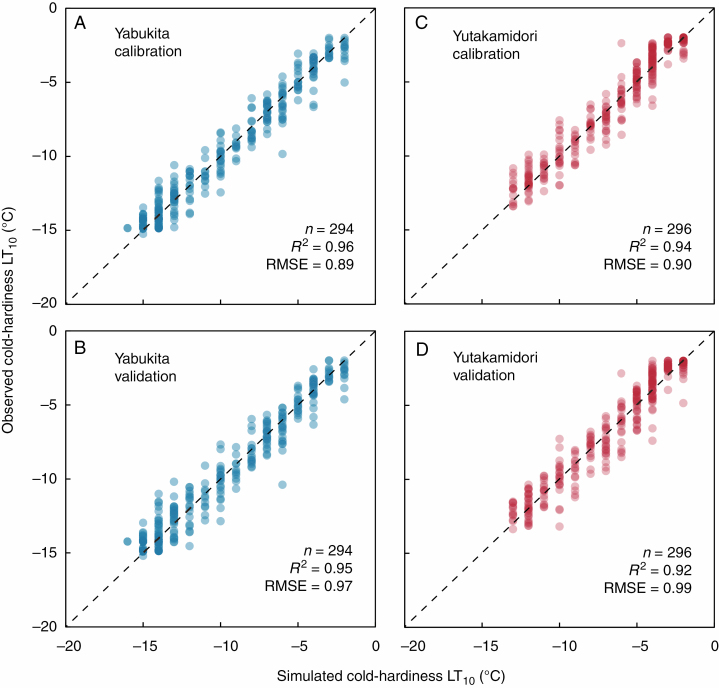
Simulated versus observed cold-hardiness (LT_10_) in the calibration and validation datasets for (A, B) ‘Yabukita’ and (C, D) ‘Yutakamidori’.

The developed model accurately predicted the genetically determined differences between the two cultivars in LT_10_ (Fig. 3), by changing only the parameters (Table 1), in both the calibration and validation datasets. The correlation analysis demonstrated that the variation in the observed LT_10_ explained 96 and 94 % of the variation in the predicted LT_10_ in ‘Yabukita’ and ‘Yutakamidori’, respectively, in the calibration dataset (Fig. 3A, C) and 95 and 92 % of the variation in ‘Yabukita’ and ‘Yutakamidori’, respectively, in the validation dataset (Fig. 3B, D). This model had high accuracy for both cultivars in the calibration datasets (RMSE = 0.89 and 0.90 °C in ‘Yabukita’ and ‘Yutakamidori’, respectively) and validation datasets (RMSE = 0.97 and 0.99 °C in ‘Yabukita’ and ‘Yutakamidori’, respectively). For the validation datasets for both cultivars, the predicted LT_10_ values were generally within 1.1 °C (90th percentile) of the observed LT_10_, even during periods when the model over- or underpredicted LT_10_.

**Fig. 4.**
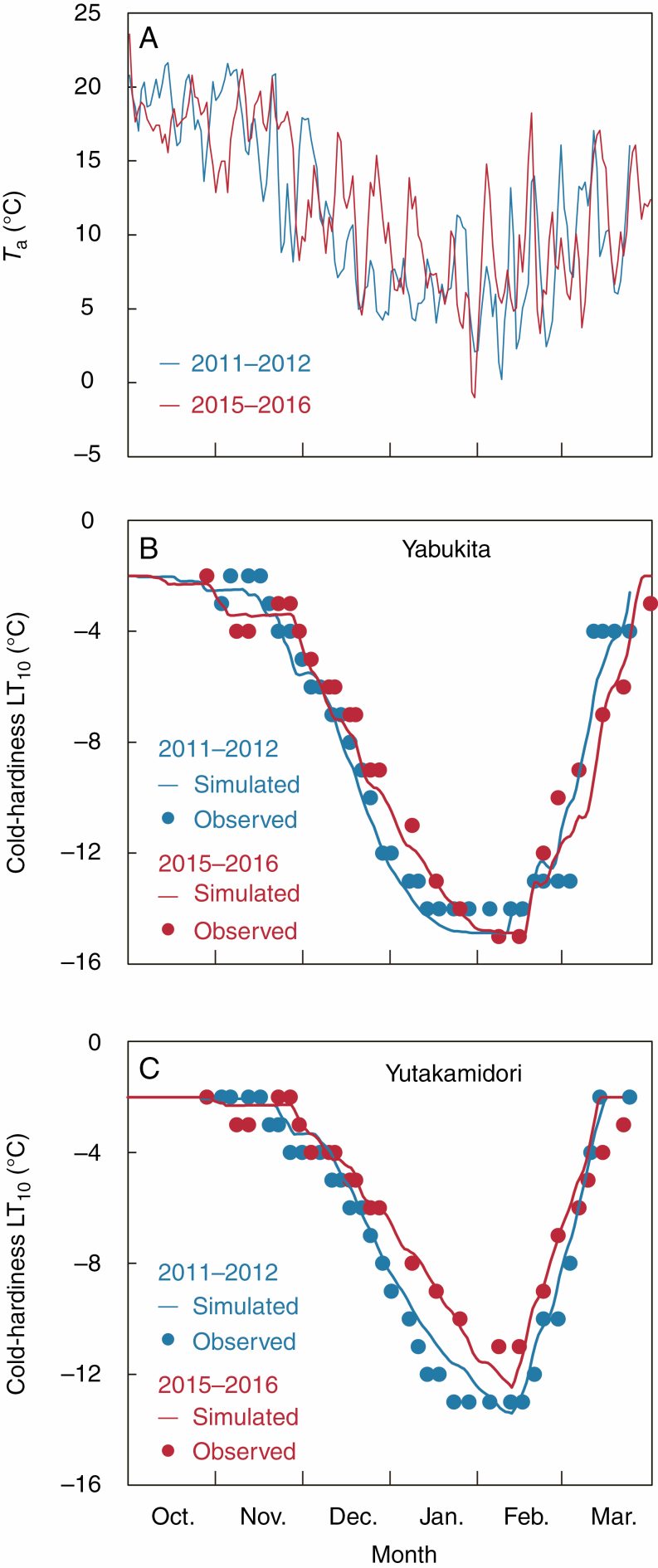
Seasonal changes in (A) daily mean air temperature (*T*_a_) and cold-hardiness (LT_10_) in (B) ‘Yabukita’ and (C) ‘Yutakamidori’ in 2011–12 and 2015–16. Simulated LT_10_ was calculated from the validation datasets.

The model simulated different seasonal changes in LT_10_ that resulted from different temperature changes (Fig. 4). Model outputs successfully reflected the delayed changes in LT_10_ in response to the warmer temperatures in the winter and the short-term changes driven by temperature fluctuations (Fig. 4); nevertheless, the changes in LT_10_ were estimated from the validation dataset (not the calibration dataset). Predictions of LT_10_ were accurate during cold acclimation (autumn to winter) but relatively less accurate after acclimation (winter to spring), owing to higher fluctuations of the ambient temperatures in spring and the shorter period of deacclimation (Fig. 4 and Supplementary Data Fig. S1). Overall, seasonal changes and cultivar differences in LT_10_ over the 10-year period were well captured by the model (Supplementary Data Fig. S1).

### Sensitivity of parameters

**Fig. 5.**
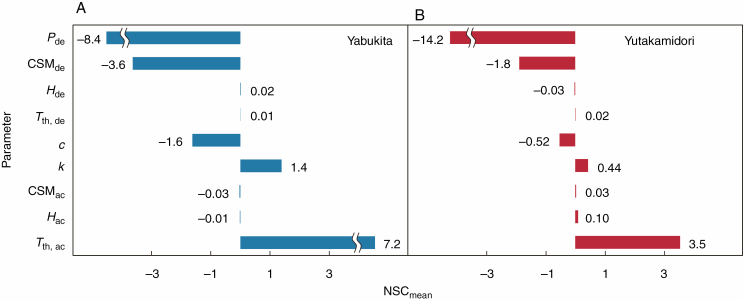
Mean normalized sensitivity coefficients (NSC_mean_) calculated for RMSE between simulated and observed values of cold-hardiness (LT_10_) in ‘Yabukita’ (A) and ‘Yutakamidori’ (B), for variations in the fitted parameters within the cold-hardiness model. The optimized value of a parameter, as shown in Table 1, was changed at 1 % intervals from –5 to +5 %, whereas all other parameters were maintained at the optimized values.

The sensitivity of the fitted parameters was similar for ‘Yabukita’ and ‘Yutakamidori’ (Fig. 5). The developed model was the most sensitive to the parameters that controlled the transition from cold acclimation to deacclimation (*P*_de_ and CSM_de_), followed by the parameters that determined cold stress memory (*T*_th,ac_, *k* and *c*). In contrast, the parameters that controlled deacclimation (*T*_th,de_ and *H*_de_) had minor effects on the model’s accuracy.

## DISCUSSION

### Model structure

We have developed and evaluated a numerical model to simulate changes in cold-hardiness in tea buds. The model was developed using our knowledge of the importance of plant memories for cold stress, and in the model these memories could be imitated by thermal time weighting, using the forgetting function (CSM in eqn 5). The use of the forgetting curve was the key to formulating CSM and developing this new model of cold-hardiness. The optimized forgetting curve showed that tea plants might develop cold-hardiness by referring to temperatures experienced during all periods of cold acclimation, and the changes in cold-hardiness were more strongly affected by temperatures in recent days than those in the previous months (Fig. 2). In plants, the accumulation of osmolytes, such as carbohydrates and proline, abscisic acid and antioxidants, which are important for the stabilization of cell vitality and increase cold-hardiness, would take more than several days to occur after plants first perceived the cold stress (Kaplan *et al.*, 2007; Baier *et al.*, 2019). Thus, the strong contributions of the recent temperatures in the model broadly reflected the synthesis of compatible solutes, antifreeze proteins and fatty acid desaturases, which are related to cold acclimation (Charrier *et al.*, 2018*b*). In contrast, the optimized forgetting curve showed that the temperatures in previous weeks and months also affected cold-hardiness (Fig. 2). This indicates that tea buds can remember cold exposures that happened weeks or months ago and use the memory to enable cold-hardiness. In plants, such long-term memories of cold stress are useful for determining the timing of budbreak and vernalization (Hilker and Schmülling, 2019), which requires a period of exposure to cold temperatures, known as the chilling requirement. In the model, the contribution of long-term temperatures mainly affected the parameters CSM_ac_, which decelerated the acclimation rate in midwinter, and CSM_de_, which was one of the factors that determined the timing of the transition from cold acclimation to deacclimation (i.e. the boundary between endodormancy and ecodormancy).

Aikawa *et al.* (2010) demonstrated that the seasonal variations in the expression of a temperature-dependent flowering-time gene in perennial *A. halleri* were explained solely by the accumulated temperatures in the previous 6 weeks. This approach was equal to that used to determine the accumulation of thermal time weighted by the rectangular function. The use of the rectangular weighting function is a reliable approach to express the expression of a single gene, but it is unclear whether the rectangular weighting is a reasonable approach to predict cold acclimation and deacclimation, which are driven by mass gene expression (e.g. Wang *et al.*, 2013 in tea plants). The forgetting curve can be interpreted as a set of rectangular functions, although actual gene expression for cold acclimation and deacclimation shows a more complex pattern. Nevertheless, the function of CSM formulated by thermal time weighted by the forgetting function enabled us to express variation of cold-hardiness in response to short-term temperatures and phenological cycles (transition from cold acclimation to deacclimation) in response to long-term temperatures.

Including the effects of both short-term and long-term temperatures in one function allowed the model parameters to be reduced. Leinonen (1996) integrated a cold-hardiness model with a phenological model (endodormancy, ecodormancy, growth and lignification) in Scots pine, and accurately predicted seasonal changes in cold-hardiness even in walnut trees, over a 3-year period (Charrier *et al.*, 2018*a*). This model is the most process-based model; however, it has several parameters (18 parameters) that were difficult to completely determine by fitting, and thus requires further field observations. In contrast, our model was formulated using almost half the number of parameters (ten parameters) by introducing the function of CSM, and it accurately predicted cold-hardiness across a 10-year period (Supplementary Data Fig. S1).

The importance of CSM was also demonstrated by the sensitivity analysis of the model parameters (Fig. 5). Four out of five parameters were related to CSM (*T*_th,ac_, *k*, *c* and CSM_de_) and were considerably sensitive to model accuracy, and among them *T*_th,ac_ was the most sensitive. The threshold temperature is linked to the criterion that plants start to accumulate carbohydrates such as starch and sugars, and the concentrations are positively correlated with the rate of cold acclimation (Greer *et al.*, 2000). In plant phenology, reference periods of past temperatures are also important, in addition to the threshold temperatures of thermal time, to detect long-term patterns of temperature changes and neglect short-term noise. The importance of reference periods has been demonstrated at the gene expression level in the flowering phenology of *A. halleri* (Aikawa *et al.*, 2010; Kudoh, 2016), and our result supports this notion with the high sensitivity of *k* and *c*, which determine the reference period and contributions of past temperatures.

Many perennial plants require a specific period of chilling to transition from cold acclimation to deacclimation (the boundary between endodormancy and ecodormancy). In this model, the boundary was expressed by CSM_de_ instead of fulfilling a chilling requirement, which has often been used in phenological modelling (e.g. Richardson *et al.*, 1974; Chuine, 2000). Generally, the strength of the chilling treatment controls budburst in many plants, including subtropical woody species, such as tea plants (Du *et al.*, 2019). This finding implies that CSM from autumn to winter probably varies the boundary (i.e. CSM_de_), but in this study CSM_de_ was assumed to be constant to simplify the model. Additionally, for budburst, subtropical plants became sensitive to the photoperiod when the chilling requirement was not met (Du *et al.*, 2019), and for leaf unfolding, species in low latitudes tended to depend on the photoperiod (Zohner *et al.*, 2016). Thus, the critical day length *P*_de_ was introduced into the model so that insufficient chilling could be offset by a longer photoperiod, and in fact the importance of *P*_de_ was demonstrated by the sensitivity analysis of the model’s parameters (Fig. 5).

Deacclimation generally occurs more rapidly than acclimation (Kalberer *et al.*, 2006), and even under the same temperature treatments acclimation and deacclimation showed different rates of change (Gay and Eagles, 1991). These findings have shown that the processes of cold acclimation and deacclimation are regulated differently but are not completely the reverse of each other. Additionally, in a combined analysis of mRNA and protein in *Arabidopsis*, sets of mRNAs transcribed under cold acclimation processes were stored until they were translated under deacclimation processes (Nakaminami *et al.*, 2014). This implies that cold stress during cold acclimation affects changes in cold-hardiness under deacclimation. The present model thus assumed that deacclimation was accelerated by forgetting past cold stresses (i.e. decreasing CSM in eqn 8) as well as the advance of growth onset by warm temperatures (increasing GDD in eqn 9).

### Model validation

We validated the model using a large dataset of seasonal changes in cold-hardiness across 10 years for two cultivars. High accuracy was noted with the model validation across the 10-year period, indicating that this model could predict the cold-hardiness of tea buds that experienced different temperature changes, raising the possibility of a successful prediction tool for cold-hardiness in future warming climates. Additionally, high accuracy in the validation for both cultivars indicates that this model can be applicable to other cultivars or close species, after adjusting the model parameters.

Charrier *et al.* (2018*a*) demonstrated that the cold-hardiness model originally developed by Leinonen (1996) could reliably predict cold-hardiness in walnut branches from three genotypes (RMSE = 2.28–2.91 °C with cold-hardiness ranging from −10 to −35 °C). Ferguson *et al.* (2011) also developed a model based on grapevine buds in three genotypes and demonstrated high accuracy of the model (RMSE = 1.43–2.27 °C with cold-hardiness ranging from −10 to −30 °C). To the best of our knowledge, there is no published model for cold-hardiness in tea plants; nevertheless, the accuracy in the present model compares favourably with those in conventional models reported, although RMSE cannot be simply compared among models because of different datasets with different ranges of cold-hardiness and input temperatures.

### Limitations of this study

Thermal time in the present model was calculated from mean daily temperatures, but the temporal resolution might not be enough to predict rapid changes in cold-hardiness in response to rapid fluctuations of the ambient temperature. Some cold responses and expressions of cold-induced transcription factors are quickly induced, within minutes or hours (e.g. Gilmour *et al.*, 1998; Vogel *et al.*, 2005; Zhao *et al.*, 2017), and some phenological developments respond to temperature differently during the day and night (e.g. circadian gating of the temperature response in flowering; Chew *et al.*, 2012). More in-depth fine-resolution understanding of the temporal changes in temperatures is thus required to develop a more reasonable process-based model.

Understanding the spatial variability of cold-hardiness is important for predicting frost damage across a wide range of regions. In this study we did not investigate whether or not the model parameters optimized here were applicable to different tea fields. Particularly the parameter CSM_de_, which determines transitions from cold acclimation to deacclimation, may vary with cold stress conditions, as discussed above. To evaluate accurate spatial distributions of cold-hardiness, a robust model of the transition from cold acclimation to deacclimation should be constructed, using a set of data observed in different fields. Additionally, fine spatial resolutions of temperature are needed to assess meaningful distributions of cold-hardiness. The model input data *T*_a,t_ had a spatial resolution of 1 km, and in this study the 1-km-grid temperatures were similar to the observed temperatures in the experimental tea field (comparison using 4 years of data; Supplementary Data Fig. S2). However, in more complex terrains, such as the hilly areas where the tea plants are usually cultivated, significant differences in temperature occur within a 1-km grid, mainly due to cold air drainage on radiative frost nights (Chung *et al.*, 2006), leading to a bias of cold-hardiness within the grid. The use of digital elevation models enables us to assess spatially the fine distribution of temperature, considering local effects of cold air drainage (e.g. Maclean *et al*., 2018), and its integration with the cold-hardiness model can provide accurate distributions of the cold-hardiness and meaningful assessments of the frost risk.

The present model is an empirical model developed based on the relationships between temperature changes and observed cold-hardiness, although the cold stress memory process of the plants was partially considered. To develop a more reliable process-based model that considers stress memories, we must experimentally understand how plants memorize long temperature exposures and at what point in the memory it is more effective for cold acclimation and deacclimation. Thus, experimental research is necessary to comprehensively understand temperature responses related to cold acclimation and deacclimation, and so are modelling studies using only observational data from natural conditions (Hänninen *et al.*, 2019).

Despite the limitations, we believe that this first attempt to develop a cold-hardiness model, using the concept of plant stress memory, will contribute to more accurate predictions of cold acclimation and deacclimation in future warming climates.

## SUPPLEMENTARY DATA

Supplementary data are available online at https://academic.oup.com/aob and consist of the following. Figure S1: seasonal changes in observed and simulated cold-hardiness (LT_10_) in ‘Yabukita’ and ‘Yutakamidori’ over 10 years. Figure S2: relationship between temperatures observed in the experimental tea field and those obtained from the Agro-Meteorological Grid Square Data.

mcaa197_suppl_Supplementary_Material
