# Supplementary material for: Dynamic modelling of cold-hardiness in tea buds by imitating past temperature memory
Source: Ann Bot. 2020 Nov 28;127(3):317–26. doi: 10.1093/aob/mcaa197 (PMC7872117; doi:10.1093/aob/mcaa197)
Supplement: mcaa197_suppl_Supplementary_Material [file mcaa197_suppl_supplementary_material.docx]

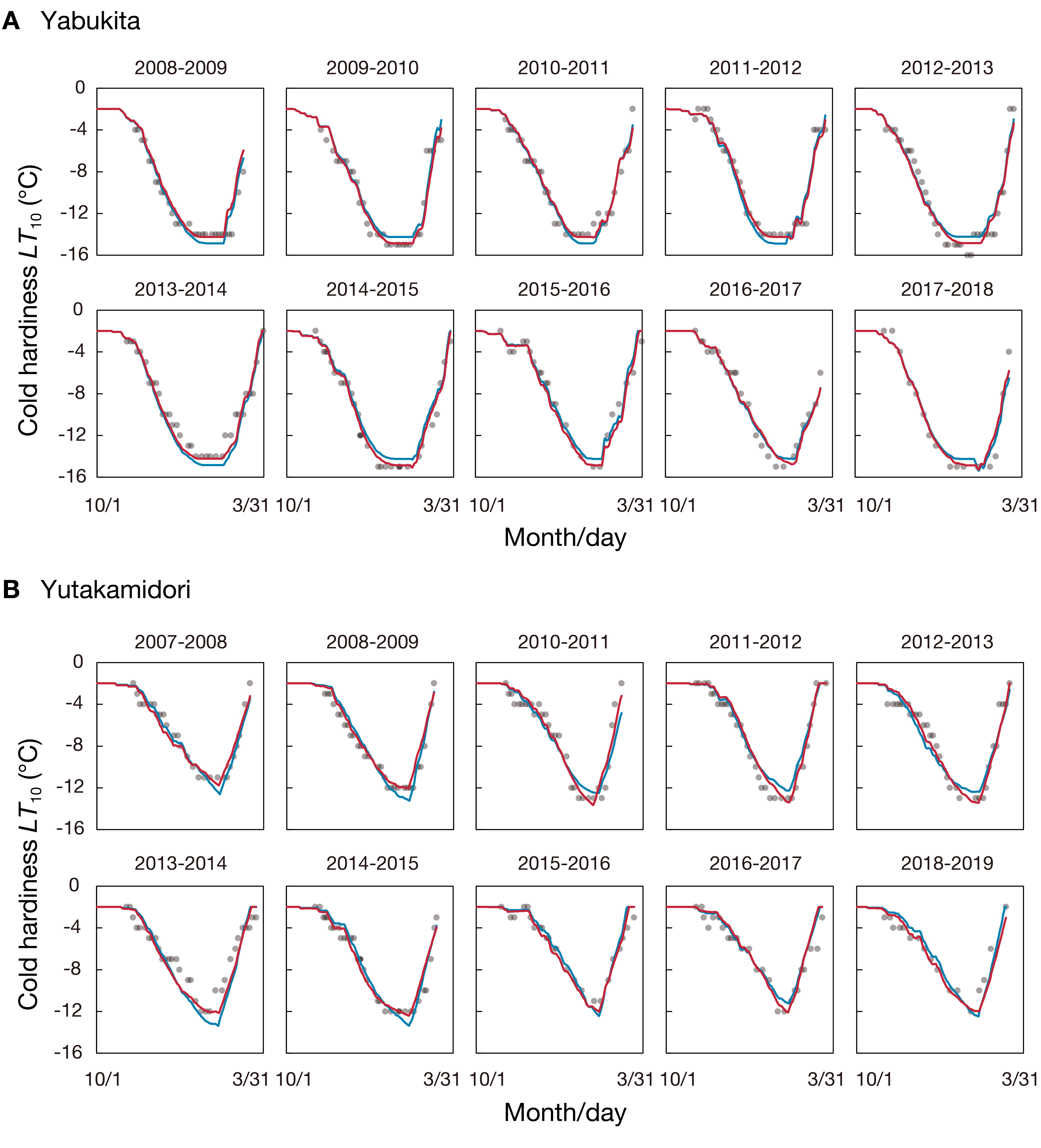


Figure S1 Seasonal changes in the cold hardiness of *LT*_10_ in Yabukita (A) and Yutakamidori (B) over 10 years. Observed values are shown in grey circles, and simulated values are indicated by red (calibration dataset) and blue (validation dataset) lines.


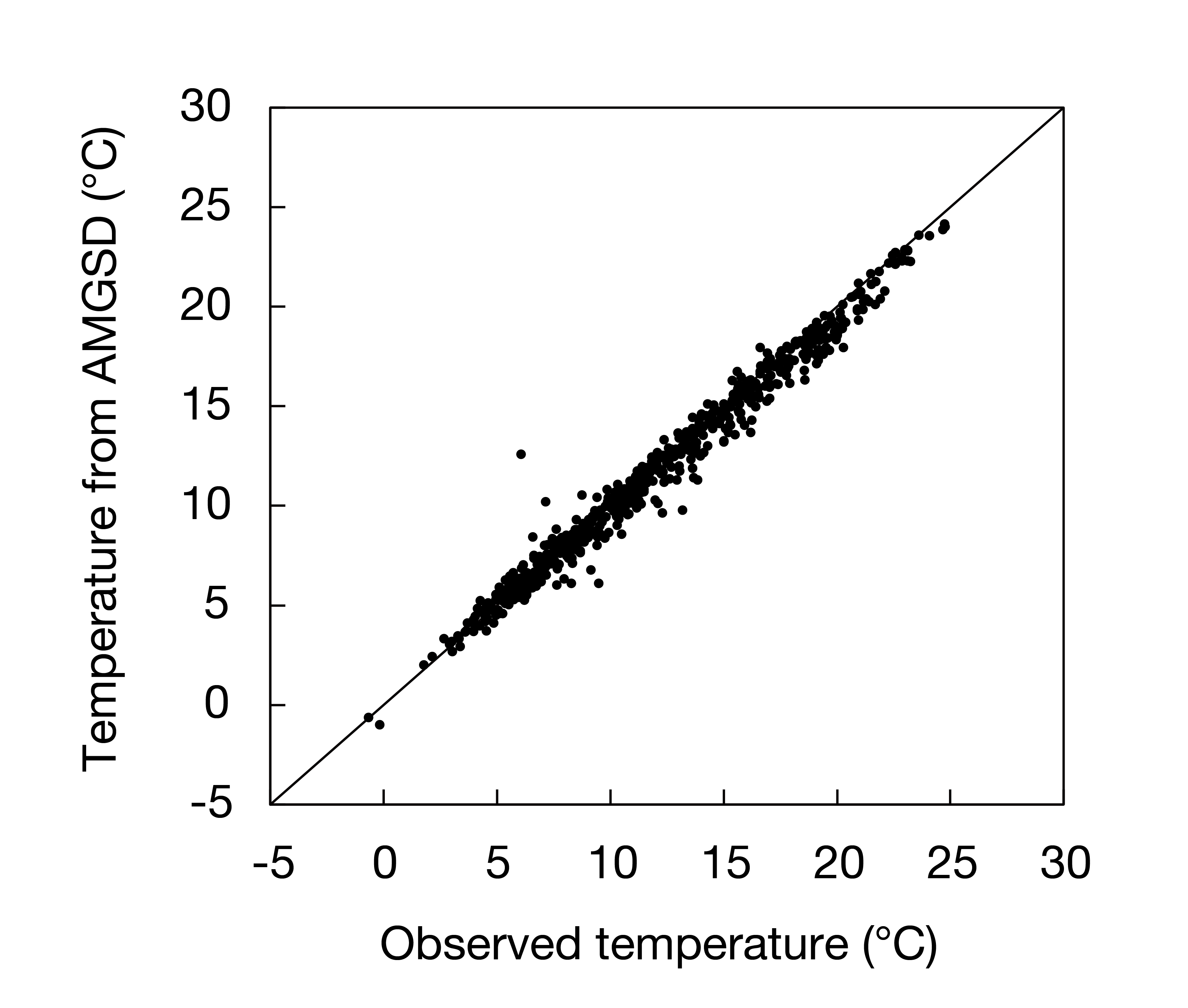


Figure S2 Relationship between temperatures observed at the experimental tea field and those obtained from the Agro-Meteorological Grid Square Data (AMGSD).
